# Supplementary material for: The Effect of Continuous Low-Intensity Exposure to Electromagnetic Fields from Radio Base Stations to Cancer Mortality in Brazil
Source: Int J Environ Res Public Health. 2021 Jan 29;18(3):1229. doi: 10.3390/ijerph18031229 (PMC7908558; doi:10.3390/ijerph18031229)
Supplement: Supplementary file 1 [file ijerph-18-01229-s001.pdf]

Supplementary Material

Results of analysis of brain cancer mortality data

In the descriptive analysis, the results show higher levels of brain cancer mortality in the last two categories of exposure to RBS (Table S1).

Table S1. Descriptive analysis of brain cancer mortality in Brazilian capitals.

	**Brain**<br>Median/10^5 (IQR)	**Sign**
**RBS**		***
**≤500**	3.53 (5.82)	
**501-1000**	4.11 (14.09)	
**1001-2000**	6.77 (18.22)	
**>2000**	6.36 (15.80)	
**Sex**		***
**Female**	5.19 (13.54)	
**Male**	6.46 (18.25)	
**Age group**		***
**< 30**	1.30 (1.28)	
**30-49**	2.73 (2.32)	
**50-69**	10.42 (9.31)	
**≥60**	28.27 (25.11)	
**Year**		NS
**2010-2011**	5.81 (15.51)	
**2012-2013**	6.33 (16.83)	
**2014-2015**	6.00 (16.56)	
**2016-2017**	5.09 (13.90)	

RBS = exposure to Radio Base Station (antennas-year); IQR = Interquartile range; Sign = statistical significance - Significance. codes: *** p-value < 0.001; ** p-value < 0.01; * p-value < 0.05; NS p-value > 0.05.

In the adjusted analysis, the results did not show higher increase in mortality rate by brain cancer for higher levels of exposure to RBS radiofrequency (Table S2).

Table S2. Adjusted risk of brain cancer mortality in Brazilian capitals.

	**Brain**	
	RR	Sign
**Fixed effects**		
**Log RBS**	0.86	***
**Sex**		
**Female**	1.00	
**Male**	1.37	***
**Age group**		
**< 30**	1.00	
**30-49**	2.43	***
**50-69**	9.55	***
**≥60**	2.53	***
**Year**		
**2010-2011**	1.00	
**2012-2013**	1.05	NS
**2014-2015**	1.10	***
**2016-2017**	9.59	NS
**Random effects**		
	Std Dev	
**Capital (intercept)**	0.87	***
**Sqrt GDP (slope)**	0.00	**
**Area/Km^2 (slope)**	0.00	NS
**Deviance**	12368	

Sqrt GDP = Square root transformation of Gross Domestic Product per capita; RR = Rate Ratio; Std Dev = Standard Deviation; Log RBS = logarithm transformation of Radio Base Station radiofrequency exposure (RBS radiofrequency exposure = sum of the number of RBS in each year multiplied by the exposure time); Sign = statistical significance - Significance. codes: *** p-value < 0.001; ** p-value < 0.01; * p-value < 0.05; NS p-value > 0.05.

Multilevel Poisson model was used to estimate the risk of cancer mortality. Except for breast and cervix cancers, which were estimated only for women, adjusted model included as fixed effects the variables sex, logarithm of RBS, age group and death year. The variables included as random effects were capital (intercept), GDP (slope) and area/Km^2 (slope). The offset of the population was also included in the models.
